# Case report: Cyclosporine A-induced extrapyramidal syndrome following hematopoietic stem cell transplantation

**DOI:** 10.3389/fneur.2023.1140732

**Published:** 2023-04-17

**Authors:** Fatema Al-Amrani, Abdulhakeem Al Rawas, Eiman Al-Ajmi, Amna Al Futaisi

**Affiliations:** ^1^Division of Neurology, Department of Pediatrics, Sultan Qaboos University Hospital, Sultan Qaboos University, Muscat, Oman; ^2^Division of Hematology, Department of Pediatrics, Sultan Qaboos University Hospital, Sultan Qaboos University, Muscat, Oman; ^3^Department of Radiology and Molecular Imaging, Sultan Qaboos University Hospital, Sultan Qaboos University, Muscat, Oman; ^4^Division of Neurology, Department of Pediatrics, College of Medicine and Health Sciences, Sultan Qaboos University, Muscat, Oman

**Keywords:** cyclosporine neurotoxicity, extrapyramidal signs, neurological adverse events, chorea, liver transplantation

## Abstract

**Introduction:**

Cyclosporine A-associated neurotoxicity has been reported in up to 40% of patients and its wide range of neurological adverse effects have been reported, ranging from mild tremors to fatal leukoencephalopathy. Extrapyramidal (EP) neurotoxicity is a rare manifestation of cyclosporine. Cyclosporine-induced extrapyramidal syndrome remains a rare adverse reaction.

**Design/methods:**

A database search was performed for studies in patients from all age groups. We found a total of 10 articles reporting EP as an adverse effect of cyclosporine A. A total of 16 patients were found, and a thorough review of these patients was performed. A comparison of patients was performed to highlight common clinical presentations, investigations during the symptomatic phase, and prognosis. In addition, we describe an 8-year-old boy who developed cyclosporine-related extrapyramidal signs on day 60 post-hematopoietic stem cell transplantation for beta-thalassemia.

**Conclusion:**

Cyclosporine A can induce neurotoxicity resulting in diverse symptoms. Signs of EP are rare manifestations of cyclosporine neurotoxicity and should be considered when evaluating post-transplant recipients of cyclosporine when they are present with any EP symptoms. Discontinuation of cyclosporine results in good recovery in most patients.

## 1. Introduction

Cyclosporine A-associated neurotoxicity has been reported in up to 40% of patients (1). This neurotoxicity can be life-threatening and necessitates dose adjustment or complete medication discontinuation (1). A wide range of neurological adverse effects of cyclosporine has been reported, ranging from mild tremors to fatal leukoencephalopathy (1, 2). The use of cyclosporine may lead to seizures, cortical blindness, pyramidal weakness, aphasia, and ataxia (1, 2). Extrapyramidal (EP) adverse effect is a rare manifestation of cyclosporine neurotoxicity (1, 2). EP side effects may include hypokinesia, orolingual apraxia, parkinsonism, severe dysphagia, and orofacial dyskinesia (3–6). Cyclosporine-induced chorea remains a rare adverse reaction.

This report describes extrapyramidal neurotoxicity induced by cyclosporine and delineates the clinical course recommended for this rare neurological manifestation.

## 2. Case

An 8-year-old boy with beta-thalassemia major underwent hematopoietic stem cell transplantation and was then given 75 mg of cyclosporine A two times daily. On day 50 after transplantation, he presented to the hematology clinic with a history of fever and was admitted for workup.

On day 60 after transplantation, he developed acute involuntary movements of his upper and lower extremities (distal and proximal) that emerge after waking from sleep and disappear during sleep.

He showed random, non-rhythmic, high-amplitude, and semi-purposeful movements. The patient's parents also reported continuous eyelid blinking when he intended to close his eyes with preserved consciousness. Moreover, he reported difficulties in walking that started roughly at the same time along with the abnormal involuntary movements. He had difficulty standing from a seated position and required assistance standing and walking. Parents reported cognitive slowness and sluggish speech.

Prior to this presentation, he underwent a normal neurological examination; all the above-listed symptoms were acute. At the onset of symptoms, he was under the following medications: folic acid, esomeprazole, levofloxacin, posaconazole delayed-release tablets, ursodeoxycholic acid capsule, and amlodipine.

**Figure 1:**
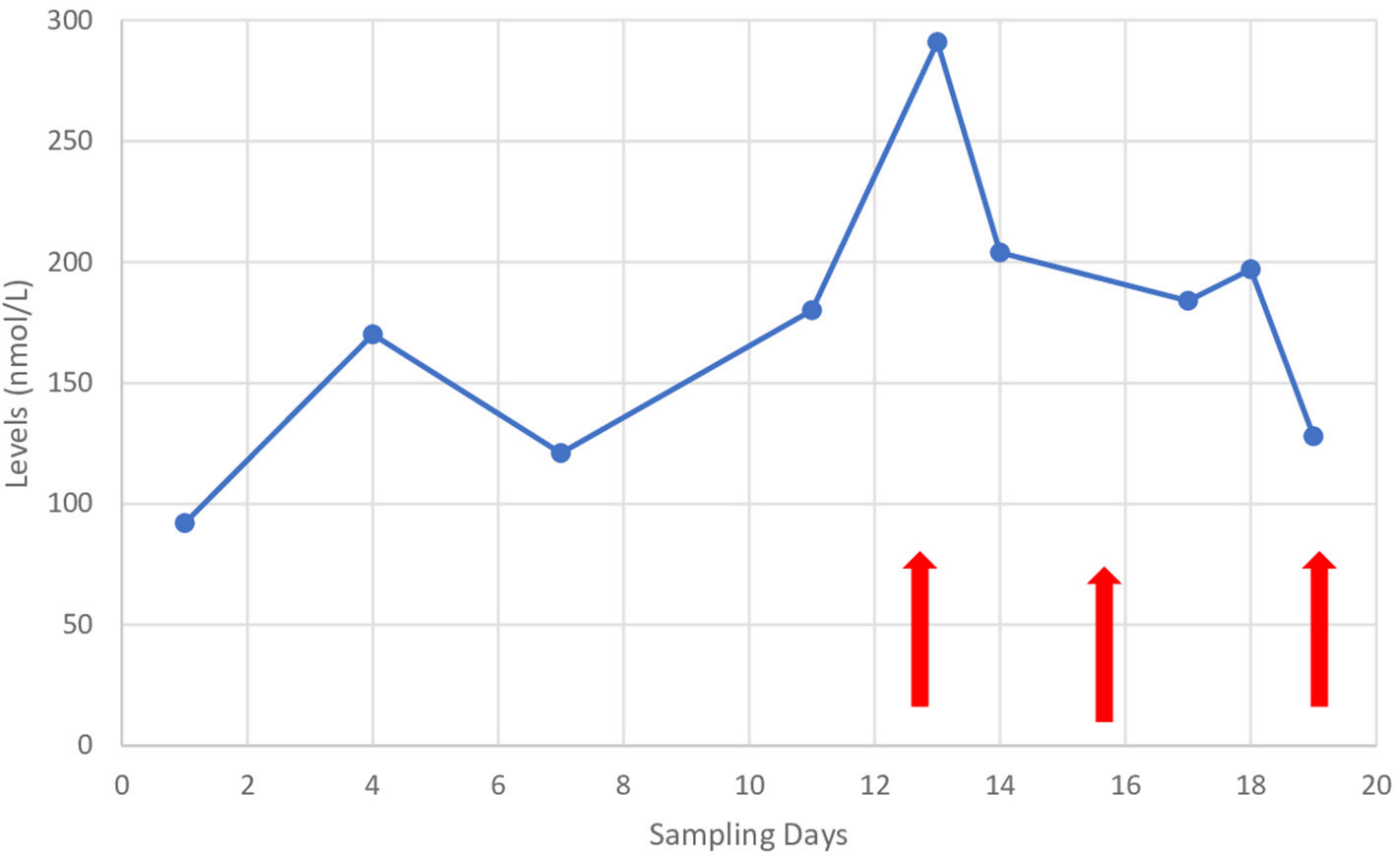
Cyclosporine levels during the patient's admission and since appearance of symptoms. Arrows on the left: indicates the onset of symptoms and middle arrow indicates cassation of cyclosporine and arrows on the right indicates disappearance of symptoms.

Physical examination showed the patient's coherence, but he responded slowly to questions. He had motor impersistence manifested during eye closure and pursuit eye movements. Hypometric saccades were observed on vertical and horizontal gaze. He had a masked face lacking facial expression with no weakness of facial muscles. Involuntary eye movements were noticed during the physical examination. He had an involuntary and abrupt eye-opening that became evident when he tried to close his eyes. Orobuccal dyskinesia was noticed during the examination and manifested in involuntary tongue protrusion in different directions, lip licking, and lip pursing. The patient had involuntary abrupt movements, involving the neck and proximal upper and lower extremities. He had slow writhing movements involving the distal upper and lower extremities. The tone examination showed rigidity in the lower extremities, normal power with no weakness, and brisk deep tendon reflexes with no asymmetry. Planters were flexors, and there was no clonus. Gait examination showed a shuffling gait with clear difficulties in movement initiation and turning during walking. The septic workup was negative as was a throat swab. Anti-streptolysin antibody titer was negative. An echocardiogram showed normal heart structures with no evidence of rheumatic heart disease. Magnetic resonance imaging (MRI) of the brain and spectroscopy was normal. Blood investigations, including electrolytes, extended lytes, liver function tests, and ceruloplasmin were all within normal limits. However, the cyclosporine level during the patients' symptoms was above the normal range (Figure 1). Cyclosporine was discontinued, and the patient's symptoms disappeared completely 3 days after cyclosporine cessation (Figure 1). Physical examination showed normal and coherent speech. There was no evidence of motor impersistence during eye closure or pursuit eye movements. The patient had normal saccadic eye movements. There was no evidence of involuntary movements of the eye, orobuccal, trunk, or extremities. Gait examination showed normal gait with no evidence of shuffling or short-step gait.

## 3. Systemic literature review

A search for patients of all ages with cyclosporine A-induced extrapyramidal syndrome in English publications (Embase, Midline, and PubMed) was performed for all years. The following Medical Subject Headings (MeSH) keywords were used: cyclosporine A, chorea, extrapyramidal syndrome, neurotoxicity, catatonia, mutism, and parkinsonism. A total of 10 articles were found. A thorough review of these articles was performed to extract data about the reported patients to examine whether an established association was found between cyclosporine A and extrapyramidal syndrome. A total of 16 patients were found to have extrapyramidal symptoms induced by cyclosporine neurotoxicity (Table 1). Data on age at presentation, patients' sex, clinical symptoms, primary disease, transplanted organ, onset of symptoms from transplant day, cyclosporine dose, cyclosporine level at the onset of symptoms, neuroimaging findings, EEG findings, day of cessation of cyclosporine, and outcome for every patient were collected. Further comparison between different parameters was performed.

**Table 1:** Summary of reported patients with cyclosporine A- induced extrapyramidal syndrome.

**Author**	**Age (yrs)/ Gender**	**Primary disease**	**Transplanted Organ**	**Cyclosporine dose**	**Cyclosporine level (nmol/L)**	**Onset of symptoms**	**Symptoms**	**Investigations**	**Day of withdrawal**	**Outcome**
Our patient	8/ M	Beta-thalassemia major	HSCT	75 mg twice daily	292	D 60	Excessive continuous blinking Orofacail/orolingual dyskinesia. Choreoathetotic movements of the limbs, neck and trunk Parkinsonism features Walking difficulties.	Brain MRI: normal ASO-titer: normal Echo: normal Throat C/S: negative Ceruloplasmin: normal	D62	Onset of improvement: D63 D65: Full recovery
Valldeoriola et al. Patient 1	36/M	HCV-related cirrhosis	Liver	NA	1418	D4	Subacute onset of dysarthria leading to mutism with normal written and oral comprehension. Severe dysphagia Focal seizures involved the Rt face Nadir: D12	Brain MRI: Normal EEG: normal CSF exam: normal. Cerebral angio: normal	D7	Onset of improvement: D15 Full recovery
Valldeoriola et al. Patient 2	56/F	HCV-related cirrhosis	Liver	NA	661	D6	Subacute onset of dysarthria leading to mutism with normal written and oral comprehension Severe dysphagia Hypokinesia and under utilization of the right UL Focal seizures involved the Rt face Nadir: D9	Brain MRI: Hyperintense GP of both lenticular nuclei in T1WI (due to chronic liver disease) EEG: diffuse slowing	D8	Onset of improvement: D10 Full recovery
Valldeoriola et al. Patient 3	55/M	HCV-related cirrhosis	Liver	NA	1109	D9	Subacute onset of dysarthria leading to mutism with normal written and oral comprehension Nadir: D16	Brain MRI: Normal EEG: Normal	D9	Onset of improvement: D21 Full recovery
Valldeoriola et al. Patient 4	58/ M	HCV-related cirrhosis	Liver	IV 4 mg/kg/day	995	D6	Speech difficulties/ complete mutism in 3 days Intact orientation & comprehension Orolingual dyskinesia Severe dysphagia required NG feeds Focal seizures involved the Rt face Nadir: D12	Brain MRI: Hyperintensity of both lenticular nuclei in T1WI (Due to chronic liver disease) SPECT: Hypoperfusion over the posterior aspect of the left frontal lobe EEG: Slowing predominantly of the left frontal lobe	D7	Onset of improvement: D19 D47: Only mild dysarthria
Bird et al. Patient 1	37/M	Chronic HBV 2° Cirrhosis	Liver	IV, 4 mg/kg/day D3 post-op	2054	D5	Mute with no spontaneous movements/ unresponsiveness to all social stimuli Oculogyric crises with upward gaze deviation.	Brain CT: normal EEG: normal	Short after symptoms	Onset of improvement: D7 Full recovery 48 hours after withdrawal
Bird et al. Patient 2	48/F	Alcoholic cirrhosis	Liver	IV, 2 mg/kg/day D2 post-op dose inc 3 mg/kg/day	0.404	D3	Mute and unresponsiveness with reduced spontaneous movements Resting tremor and increased append tone Severe orofaical dyskinesia with tongue protrusion and grimacing Choreoathetoid movements of the arm Three generalized seizures 7D after withdrawal: Trismus and inability to swallow.	CSF exam: normal with negative serology EEG: diffuse slowing Brain CT: mild atrophy in keeping with alcohol abuse No focal abnormalities MRI-brain: Focal areas of T2 hyperintensities in the pons MRI-brain (12 m post transplant): showed no abnormalities in the pons	D3	Onset of improvement: after 48 hours Slowly improved but required NG-feed for few days 6 weeks post-transplant: Involuntary movements of the tongue, spasticity of the tongue with mild dysphonia and mild cerebellar ataxia
Bird et al. Patient 3	53/F	Primary biliary cirrhosis	Liver	IV, 2 mg/kg/day D6	0.204	D8	Withdrawn & mute with little motor activity with little reaction to sensory stimuli Increasing difficulty in swallowing requiring nasogastric tube feeding Pseudobulbar palsy with weakness and immobility of the palate and the tongue causing severe dysphageia and dysarthria. Mild cerebellar ataxia.	CSF exam: normal EEG: Diffuse slowing Brain CT: normal MRI brain: focal areas of increased T2 signal intensity in the mid pons in T2WI compatible central pontine myelinolysis	D8	Onset of improvement: D14 Symptoms resolved after 4 weeks
Garcia et al.	68/M	Diffuse Coronary heart disease	Heart	Oral, 75 mg/day	351	19 years	Chorea like abnormal involuntary movements Cognitive impairment Progressive walking difficulty.	Brain MRI: Ischemic foci, microhemorrhage and periventricular hyperintensity probably due to chronic ischemic changes Brain CT: Cortical and subarachnoid atrophy	19 m after onset	Regain ability to walk Regain sphincter control Didn't regain his lost cognitive function
Combarros et al.	23/M	Wilson disease/ Portal hypertension	Liver	140-200 mg twice. Daily D21 post-op: CA dose: 525 mg twice Daily	1793	D31	Continually grimacing, blinking, chewing & showing bizarre movements of the mouth and tongue Slurred speech Orofacial dyskinesia Continuous rotation movements of the neck Choreic movements of the hands.	Brain MRI: No new changes in the MRI with stable T2 hyperintensities in the putamina due to Wilson disease	Shortly after symptom onset	Involuntary movements gradually disappeared over next 10 days
Heekin et al.	9/M	Nephrotic Syndrome due to focal segmental glomerulonephritis	No transplant	125 mg am, 100 mg qHS	3892 Just before onset of symptoms 203 At the peak of his symptoms	NA	Prolonged staring, mutism, unusual arm posturing, insomnia, and abnormal gait Refuse to swallow, repeatedly spitting out his medication Sleep disturbance, episodic agitation & confusion Not following commands, while at times displaying echolalia and verbigeration Progressively agitation, 24 hours later, became Cotatonic with delusion, telling his parents he had died from “a bomb in his neck” and that he was now a “zombie followed by increasing mutism and odd mannerisms Autonomic symptoms.	Brain MRI: mild-to-moderate prominence of cerebrospinal fluid (CSF) spaces and mild thinning of the corpus callosum	NA	Onset of improvement: D39 Symptoms resolved completely on D68
Miyagi et al.	42/M	HCV-related cirrhosis	Liver	200 mg per day	NA	D8	Rest tremor Parkinsonism	NA	D16	Onset of improvement: NA Symptoms resolved completely on D75.
Lima et al.	51/M	Acute lymphoblastic Leukemia	HSCT	3 mg/kg/day	WNL. (Exact level NA)	D60	Rest tremor, reduced facial expression, dysarthria, bradykinesia, rigidity & reduced arm swing	MRI-brain: small bilateral subdural hemorrhage that was present previously	After D60 (Exact day NA)	Onset of improvement: NA Symptoms resolved completely on D120.
Kim et al.	41/F	End stage renal disease 2° to chronic glomerulonephritis	Renal transplant	300 mg per day	WNL (Exact level NA).	D36	Severe neuralgia, dysarthria, brdaykinesia followed by dyskinesia few days later Couldn't turn her body without the help of others, examination showed Parkinsonism features with rest tremor, reduced facial expression and limb rigidity.	MRI-brain: bilateral high SI in basal ganglia FU CT-brain (3 m from onset of symptoms) showed no abnormality in the basal ganglia	D69	Onset of improvement: D83 Symptoms took 3 months for full resolution.
Ling et al.	59/F	End stage renal disease with inconclusive etiology	Renal transplant	150 mg per day	195	NA (at least 4 yrs)	18 of progressive bradykinesia required assistance with walking and feeding. 10 months from symptom onset: stuporous and bed ridden Akinesia, severe rigidity, rest tremor and myoclonus, cervical dystonia with anterocollis and laterocollis	Brain MRI: minimal T2 hyperintensities in the periventricular white matter, MRA: normal	NA	Onset of improvement: within 4 days from discontinuation Symptoms took 5 months for full resolution (remained with residual ataxia).
Miklavcic et al. Patient 1	17/M	Paroxysmal nocturnal hemoglobinuria and CVST	HSCT	NA	WLN (Exact level NA)	D19	Trembling hands, difficulties using the right arm Exam: hypomimia, hypophonia, right hand rest tremor, bilateral rigidity, bradyhypokinesia & rt upper limb dystonia 4 months later: repetitive slow dystonic contractions of the Rt side	MRI-brain: asymmetric bilateral T2/FLAIR hyperintensities in the in putamen and caudate nuclei with areas of diffusion restriction in keeping with atypical PRES, MRA: normal CSF exam: normal with negative serology Dopamine transported imaging (^123^I-Ioflupane SPECT): no evidence of presynaptic dopaminergic deficit MRI-brain (4 months later):BG atrophy with less pronounced signal changes Brain FDG/PET-CT: Bilateral BG hypermetabolism.	After onset of symptoms.	No marked improvement At 1.5 yrs: mild dystonia and parkinsonism
Miklavcic et al. Patient 2	51/F	Dissiminated plasmacytoma	HSCT	NA	WLN (Exact level NA)	D47	Decrease responsiveness & unusual movements of her Rt arm 10 days later, encephalopathic with agitation and confusion Exam: disoriented, hypomimic, bilateral postural tremor, bilateral rigidity & bradykinesia with marked dystonia of both hands & extensor planters	MRI-brain: T2/FLAIR hyperintensities in bilateral occipital lobe, cerebellum, thalamus, and BG, consistent with PRES, MRA: normal CSF exam: normal with negative serology	After onset of symptoms.	Died soon after from necrotizing cholecystitis caused by gallstone

M, male; F, female; yrs, years; UL, upper limb; HSCT, hematopoietic stem cell transplantation; ASO, anti-streptolysin antibodies; C/S, culture; HCV, hepatitis C virus; NA, not available; IV, intravenous; D, day; NG, nasogastric; MRI, magnetic resonance imaging; MRA, magnetic resonance angiography; CT, computed tomography; T1WI, T1 weighted image; DWI, diffusion weighted image; SI, signal intensity; SPECT, single-photon emission computerized tomography; EEG, electroencephalogram; CSF, cerebrospinal fluid; Rt, right; am, at morning; qHS, at bed time; Cyclosporine reference range, 80-300 nmol/L; FU, follow-up; CVST, cerebral venous sinus thrombosis; PRES, posterior reversible encephalopathy syndrome; BG, basal ganglia; FDG/PET, fludeoxyglucose/position emission topography.

## 4. Discussion

Extrapyramidal manifestations are rare adverse events of cyclosporine A neurotoxicity, which includes a spectrum of symptoms ranging from akinetic mutism to tremors (1, 2). However, reported patients are scarce, and the symptomatology timing, neuroimaging findings, and recovery after the discontinuation of medication are not well understood (3–10). A total of 17 patients are reported in the literature with cyclosporine neurotoxicity-induced EP signs, including our patient (3–12). The clinical symptoms of these patients can be devastating and can include severe functional impairment. These extreme clinical symptoms have been reported in 10 of 17 patients with mutism and reduced spontaneous movements lasting for at least a few days (3, 4, 8, 9, 11) (Table 1). Seven out of 10 patients were reported to have normal written and oral comprehension as well as intact orientation (3). In contrast, three patients were reported to have some encephalopathy (one with agitation and odd mannerism followed by mutism, one with stuporous and bedridden 10 months after symptom onset of progressive dyskinesia, and one with reduced responsiveness and abnormal movements of the right arm) (8, 9, 11). Among the 10 patients with mutism, five started with speech difficulties/dysarthria and progressed to complete mutism with decreased spontaneous movements. Moreover, five of 10 patients who had mutism developed severe dysphagia and swallowing difficulties that necessitated nasogastric tube insertion for feeding (3, 4) (Table 1). Although these symptoms seemed to be present in most patients (10/17), none of these symptoms were present in our patients. Nonetheless, the striking presentation with predominantly bulbar symptoms is of interest. It is hard to speculate based on this case series whether cyclosporine neurotoxicity has more predilection to accumulate in the anterior cingulate gyrus and striatum, leading to effects on the neuro-circuit and subsequently resulting in this constellation of symptoms. This may be supported by the single-photon emission computerized tomography hypoperfusion over the posterior aspect of the left frontal lobe in one of the affected patients (3). Furthermore, these symptoms could be due to direct toxic effects at the level of the basal ganglia based on two patients reported to have signal changes in the basal ganglia (Table 1) (8, 10). However, it is hard to conclude due to the small number of patients in this study. Moreover, the EP adverse effect could be due to the cyclosporine A direct neurotoxic effect on the cell function at the level of neurotransmitters by modulating dopaminergic transmission in striatal neurons (10). Our patient had continuous eye blinking that was exacerbated when the patient intended to close his eyes, and it disappeared during sleep. This interesting phenomenon was reported previously (6). In addition, our patient had the orofacial/lingual dyskinesia present in the previously reported three patients; this did not appear to be universal in all patients (3, 4, 6). Moreover, four of 17 patients had choreoathetosis involving limbs, including our patient (4–6). This choreoathetosis was severe in our patient and caused impairment in his daily activities. One patient had an oculogyric crisis (4). Six of the 17 patients were reported to have parkinsonism (7–10, 12). Parkinsonism symptoms vary from rest tremors to severe rigidity, dyskinesia, and unresponsiveness. Our patient exhibits some parkinsonism features at his presentation manifestation such as reduced facial expression, bradykinesia, rigidity, and abnormal shuffling gait. Four of 17 patients were reported to have seizures along with the EP signs (focal seizures [3/17] and generalized seizure [1/17]) (3, 4). Only two patients had mild cerebellar ataxia (4) (Table 1).

Our patient's clinical symptomatology and parkinsonism signs and symptoms were very similar to those of the patient reported by Combarros et al. (6). Whether the association is related to the young age in both patients compared to the older age that presented with akinetic mutism/severe bulbar symptoms is unknown. It is unclear whether it has an association with cyclosporine neurotoxicity predilection to accumulate in different brain tissues in children compared to the adult/older population. It is hard to speculate based on this small case series, but this is an interesting observation that deserves further systemic evaluation in future.

Most patients (9/17) underwent liver transplantation. Our patient is the youngest pediatric patient to develop symptoms post-hematopoietic stem cell transplantation. There does not appear to be a specific dose at which patients develop neurotoxicity. Seven of 17 had a supratherapeutic level of cyclosporine at the onset of symptoms. In contrast, two patients had a subtherapeutic cyclosporine level. Seven of 17 patients had it within the therapeutic range and one with no reported cyclosporine level (Table 1). Therefore, EP signs could be triggered by high therapeutic levels. There are, however, likely other risk factors that also play a role in developing cyclosporine neurotoxicity. Moreover, the cyclosporine level at the symptomatic phase might not reflect the exact neurotoxicity that could be related to cyclosporine metabolites (9). Eight of 17 patients developed symptoms within 3–30 days post-transplantation. In addition, five patients developed symptoms after D30 but before D60 from the transplant. Data were not available for one patient. Two patients developed symptoms at least 4 to 19 years post-transplantation (5, 9). The authors speculate that this delayed presentation of cyclosporine neurotoxicity may be due to accumulated neurotoxicity over the years (5).

Sixteen patients of 17 underwent neuroimaging during the symptomatic phase, and neuroimaging was not available for one patient. Neuroimaging during the symptomatic phase showed abnormal MRI findings in 12 patients (six with basal ganglia (BG) abnormalities, two with central pontine myelinolysis, one with non-specific ischemic changes, one with non-specific changes in prominent cerebrospinal fluid spaces and corpus callosum thinning, one with bilateral subdural hemorrhage that was present previously, and one with non-specific T2 hyperintensities in the periventricular white matter). Normal MRI was reported in four patients (Table 1). Among the six patients with BG abnormalities, two were due to chronic liver disease, one was unchanged Wilson disease abnormalities, two were in the context of MRI changes that were compatible with posterior reversible encephalopathy syndrome (PRES), and one was reported to have BG changes highly consistent with parkinsonism (3, 4, 8–10). BG involvement was reported as an uncommon pattern in PRES (13). Miklavčič et al. reported that both patients had MRI changes that were compatible with PRES (8). This is of interest as hypertension was proposed as a possible mechanism for cyclosporine neurotoxicity (2). This might result in the disruption of the blood–brain barrier and involvement of the BG and subsequent direct toxic effect on the BG and development of clinical symptomatology. However, it is hard to have a conclusion based on observation in only two of the 17 patients (8). Furthermore, one patient from the six reported with BG involvement, one had reversible BG changes reported as high signal intensity and the author stated that it is highly consistent with Parkinsonism (10). However, it was unclear which sequence and which specific nuclei were affected by the high signal intensity. Furthermore, parkinsonism is a symptom of many underlying etiologies. Therefore, the neuroimaging findings depend on the underlying etiology. Moreover, central pontine myelinolysis was proposed as a pattern of neuroimaging finding in patients with cyclosporine A-induced EP syndrome. This pattern was, however, seen in two of the 16 patients, and it is likely a coincidental finding rather than a consistent pattern related to cyclosporine neurotoxicity. There is probably no consistent MRI pattern associated with cyclosporine neurotoxicity *per se*. Establishing a specific MRI pattern in cyclosporine neurotoxicity will require larger scale studies. An electroencephalogram (EEG) was performed on six patients. Three had an abnormal EEG with slowing and three had a normal EEG. All patients with an abnormal EEG had seizures during the symptomatic phase. A cerebrospinal fluid examination was performed in three patients; the results were normal in all of them, with no reported abnormalities. All the investigations performed on our patient including brain MRI, echocardiogram, ASO titer, and throat cultures were negative.

Our patient had a full recovery and fully regained his function after the cessation of cyclosporine. Overall, the prognosis seems to be good in cyclosporine neurotoxicity associated with EP signs. Eleven of 17 patients had a full recovery with no symptoms after the cessation of the medication (Table 1). Five patients continued to have symptoms after the discontinuation of cyclosporine (one of five patients had mild dysarthria, one of five patients had involuntary movements of the tongue with mild dysphonia and cerebellar ataxia, one failed to regain his full cognitive function, one had residual ataxia, and one had dystonia with mild parkinsonism) (3–5, 8, 9) (Table 1).

The time to complete recovery varied from 2 to 90 days (Table 1). However, the onset of improvement was achieved within a short period from the time of discontinuation in most patients (2–47 days from discontinuation).

Cyclosporine predispositions for neurotoxicity are due to multiple risk factors, none of which our patient seemed to have (2). The mechanism by which cyclosporine causes neurotoxicity that leads to a wide spectrum of symptoms has been an active area of research. The exact mechanism of neurotoxicity is not well understood. There are, however, several hypotheses explaining this possible mechanism. One proposed hypothesis is that the inhibition of 26-hydroxylase activities in hepatocytes, in addition to the inhibition of chenodeoxycholic acid synthesis, leads to the accumulation of abnormal sterols. Subsequently, this results in the disruption of the blood–brain barrier and accumulation in the central nervous system cells. Among the three discussed hypotheses, this hypothesis could explain our patient's symptoms. The second hypothesis proposed hypertension, hypomagnesemia, or renal insufficiency as a possible mechanism for neurotoxicity; however, our patient did not have any of these factors. The third hypothesis proposed vasculopathy or any vascular complications as a possible mechanism for neurotoxicity, such as drug-induced brain microangiopathy, similar to hemolytic uremic syndrome (9). Our patient did not show any symptoms of hemolytic uremic syndrome. It is unlikely that the last two hypotheses explain cyclosporine neurotoxicity in our patient (2). Although this damage is temporary in most reported patients, it can be permanent in others (4, 5). The difference in long-term outcomes is not yet understood. However, there is evidence that cyclosporine A induces neuronal apoptosis and selective oligodendrocyte death (13). Other factors that might cause permanent damage have yet to be clarified.

It is difficult to speculate whether one mechanism or all mechanisms contributed to cyclosporine neurotoxicity in our patient. Among the three proposed mechanisms, however, the first hypothesis seems to explain our patient's symptoms the best.

In conclusion, cyclosporine A can induce neurotoxicity resulting in diverse symptoms. Signs of EP are rare manifestations of cyclosporine neurotoxicity and should be considered when evaluating post-transplant recipients of cyclosporine when they are present with any EP symptoms.

## Data availability statement

The datasets presented in this article are not readily available because of ethical and privacy restrictions. Requests to access the datasets should be directed to the corresponding author.

## Ethics statement

Ethical review and approval was not required for the study on human participants in accordance with the local legislation and institutional requirements. Written informed consent from the participants' legal guardian/next of kin was not required to participate in this study in accordance with the national legislation and the institutional requirements. Written informed consent was obtained from the participant/patient(s) for the publication of this case report.

## Author contributions

All authors listed have made a substantial, direct, and intellectual contribution to the work and approved it for publication.
